# The New Normal: Coronavirus Pandemic Response Utilizing Microsoft SharePoint

**DOI:** 10.1007/s10278-021-00419-4

**Published:** 2021-03-10

**Authors:** Corey J. Hiti, Jennifer Chang, Kriti Gwal, Eva Escobedo, Margaret Rea, Jasjeet Bindra

**Affiliations:** UC Davis Medical Center, 4860 Y Street, Suite 3100, CA 95817 Sacramento, USA

**Keywords:** Graduate medical education, Microsoft SharePoint, Radiology, Residency, COVID-19

## Abstract

On March 11, 2020, the World Health Organization (WHO) declared the novel coronavirus disease (COVID-19) a pandemic. Hospitals throughout the USA and the residency programs housed therein have drastically altered their standard operations to slow the spread of disease. Maintaining the educational mission of a residency program within the parameters of social distancing mandates is an unprecedented challenge to graduate medical education and will remain so for the near future. We evaluated resident perception of our efforts to transition educational activities within the residency to an online, remote format utilizing Microsoft SharePoint. Educational conferences were transitioned to a videoconferencing platform on March 13, 2020 in conjunction with the deployment of an intradepartmental Microsoft SharePoint site to provide educational resources, disseminate scheduling changes, and provide wellness resources in response to the COVID-19 pandemic. An 11-question survey available from 4 Jan 2020–4 Jun 2020 surveyed resident perceptions of the instituted changes. Twenty-six of 31 residents (83.8%) responded to the resident survey invitation. Twenty-four of 26 residents (92.3%) characterized the SharePoint as useful. Twenty of 26 residents (76.9%) desired attending guidance as to how to utilize the online resources for each resident rotation. A total of 92.3% (24/26) of residents felt that the program’s response utilizing SharePoint engendered a greater sense of belonging to their work community. Resident perception of the transition to online learning utilizing Microsoft SharePoint as a distribution platform was generally positive, helping to mitigate untoward educational consequences of the COVID-19 pandemic.

**Supplementary Information:**

The online version contains supplementary material available at 10.1007/s10278-021-00419-4.

## Background

Since the beginning of the coronavirus disease 2019 (COVID-19) pandemic, residency programs have had to respond to the consequences of widespread social-distancing practices enacted to “flatten the curve” and reduce spread of disease [1–3]. Resident rotation schedules have been re-arranged to provide “surge” staffing and minimize resident exposure within the hospital. Elective procedures have been rescheduled, while COVID-19 cases themselves have had a geographically variable impact on hospitals. Although promoting public safety and slowing the spread of the COVID-19 pandemic remains the priority at most academic institutions, these changes represent a significant disruption of the traditional methods of teaching within most residency programs.

The “new normal” for residency programs involves a more exclusive reliance on remote learning and online resources to fulfill the educational role entrusted to them by the Accreditation Council for Graduate Medical Education [4]. The incorporation of an online curriculum into residency training is not a new phenomenon, with residency programs across multiple specialties reporting success in the literature [5–8]. However, the dissemination of an online curriculum has taken various forms, such as subscriptions to commercially available curriculum websites [5], embedded curriculum materials on tablets distributed by the residency [8], and web-based course management systems for residency-generated curricular materials [6, 7]. These varying methods of distribution include intrinsic differences in financial costs, portability, access, and design customization for individual programs.

We report herein our development of an intradepartmental Microsoft SharePoint site for the distribution of online materials in response to the COVID-19 pandemic within an academic radiology program. Included in the Microsoft Office 365 suite of applications used by our university and numerous others [9], SharePoint allows for the creation of intranet sites that can be managed by end-users within the enterprise. Its capabilities include, but are not limited to, document storage, interactive message boards, customized e-mail distribution, and hyperlink sharing [10]. As an enterprise-level solution, there were no direct costs to the department incurred by the creation of our site, and it leveraged other institutional benefits such as hosting on internal institutional servers and secure logins utilizing the same credentials/dual-factor authentication as other secure university sites. Given its benefits, likely widespread availability at other institutions, and ease of use, we aimed to evaluate resident perception of SharePoint for distribution of educational content, wellness resources, and scheduling changes as we responded to the COVID-19 crisis. We hypothesized that most residents would appreciate this new method of distributing educational material and the flexibility it afforded.

## Methods

Following approval from the local information technology (IT) department, a basic SharePoint site with a default template was created on our institutional intranet within a few minutes of our request. The SharePoint site was customized and designed by the Education Chief Resident within our program, who serves as a resident liaison for curricular changes and had no formal computer science or web design background prior to designing the SharePoint site. As a “site owner” (the highest level of privilege afforded to end-users within SharePoint), our Education Chief Resident was able to assign key members of the residency leadership team similar site privileges (Chief Residents, Program Directors, and Vice Chair of Education) allowing for site design modifications. Additional faculty point-persons within each section were given editing capabilities to post and curate educational content. All residents, fellows, and faculty were given permission to view and download posted resources.

Online resources, many of which had been made recently available in response to the COVID-19 crisis from subspecialty societies [3], were posted within SharePoint within a document library. The document library allowed posts of various media in the form of videos, lecture slides, and hyperlinks to relevant websites. The department-wide transition to videoconferencing, made possible by enterprise accounts our university had with both the Cisco WebEx and Zoom videoconferencing platforms, led to a proliferation of our own internal educational content in the form of recorded lectures which could be distributed and stored effectively via the SharePoint website for future use. The site also served to distribute weekly schedules for staffing modifications related to the COVID-19 pandemic utilizing an embedded Microsoft Excel document with permissions set such that program leadership could edit the document while residents and staff could view these updates immediately. As a morale-boosting endeavor, a webpage was created within the SharePoint site with a SharePoint “Photo Library” app to host a coronavirus meme contest. Additional wellness resources were posted to support residents and staff in these trying times [11].

Site usage statistics, automatically tracked by Microsoft SharePoint, were extracted for the dates Mar 13, 2020–May 1, 2020. An 11-question survey (Supplemental Content) was distributed to the residents to assess their perception of the program’s response to the COVID pandemic and possible future modifications to the curriculum. The survey was deemed exempt by the institutional review board. Nine of the questions utilized a 5-item Likert scale, while two of the questions were yes/no. The first five questions centered on aspects of the videoconferencing experience that had been rolled out for noon-time conferences and for the purposes of this study were excluded from further analysis. The next three questions centered on the electronic distribution of learning resources on the SharePoint site. The last questions centered on resident perception of wellness initiatives, rescheduling efforts, and overall feeling of community during the department’s response. The residents were given 6 days (4/1–4/7) to respond to the survey. Compilation of the survey results was performed using Qualtrics.

## Results

**Fig. 1:**
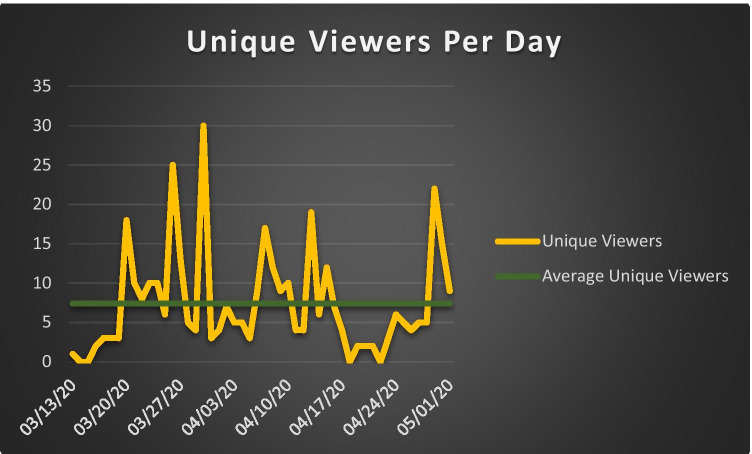
Graph plotting the number of unique viewers of the SharePoint site per day in the 50-day time period extending from Mar 13, 2020 to May 1, 2020

**Fig. 2:**
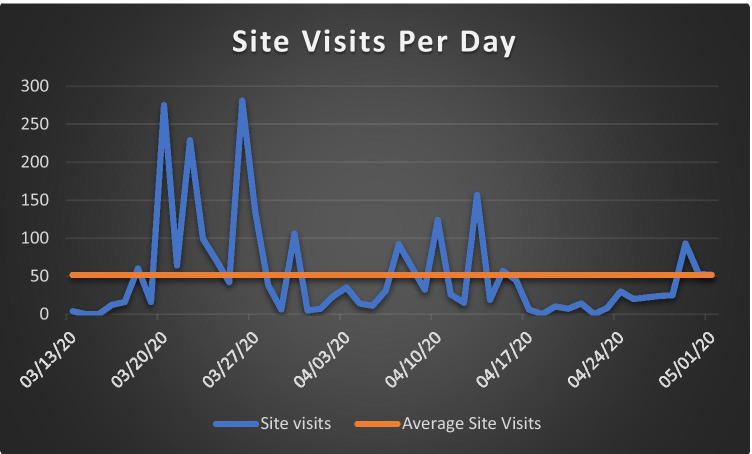
Graph plotting the number of site visits per day to the SharePoint site in the 50-day time period extending from Mar 13, 2020 to May 1, 2020

Over the study period, the SharePoint site logged 71 unique users, out of a potential 90 users (78.9%) from within our residency program. Of the potential users, 31 were residents, 10 were fellows, and 49 were faculty or staff. Site usage for the first 50-day period of the SharePoint site (Mar 13/20–May 1, 2020) recorded 51 average site visits per day and an average of 7 unique users per day. The greatest number of site visits on a given day was 281, and the greatest number of unique users was 30 (Figs. 1 and 2).

**Fig. 3:**
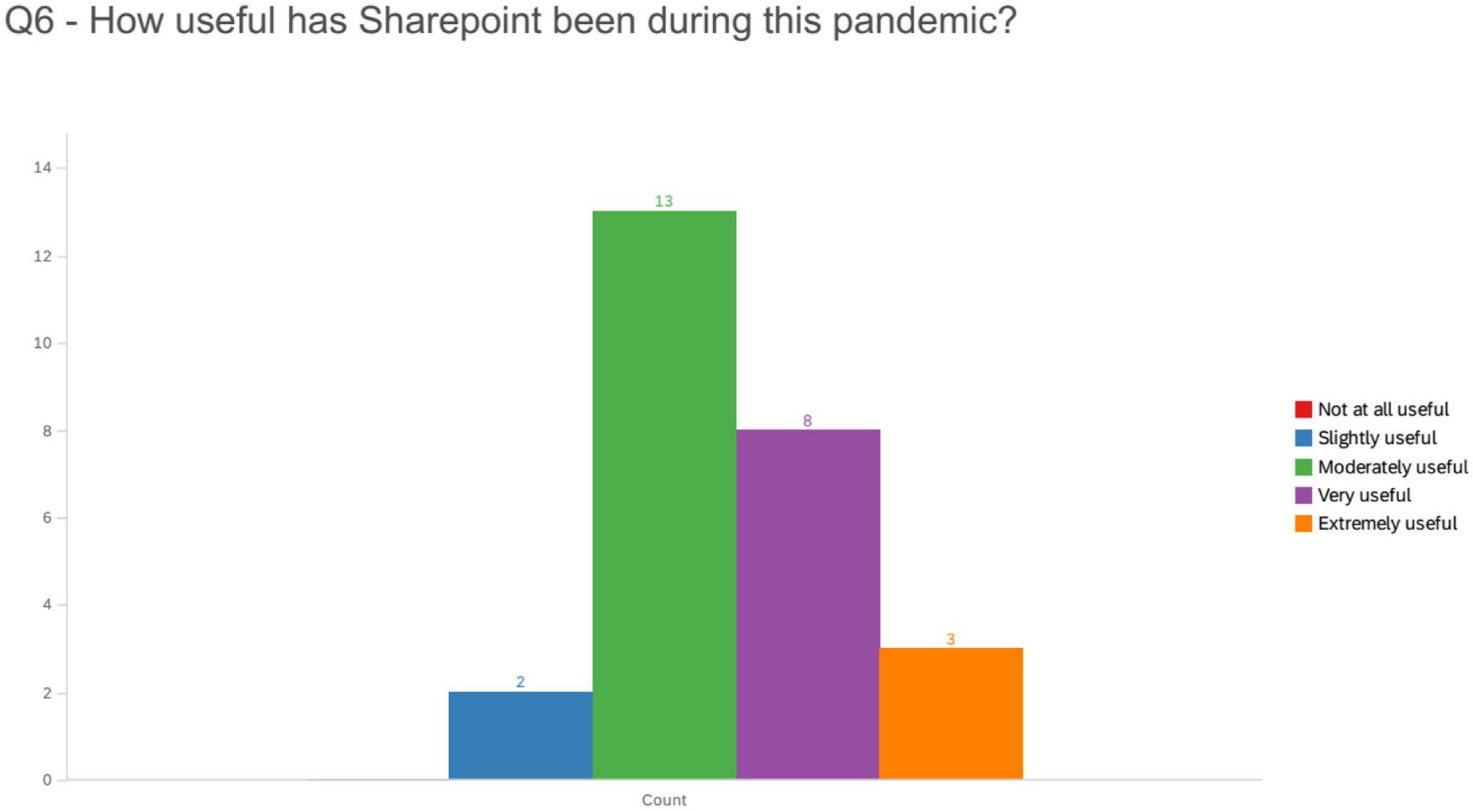
Resident survey responses regarding the utility of SharePoint

Out of the 31 residents included on the resident survey invitation, 26 responses were recorded, representing a response rate of 83.8%. Resident responses regarding the use of the SharePoint site were positive overall. Nearly all residents felt that the SharePoint website served some utility, with 80.8% (21/26) feeling it was moderately or very useful and an additional 11.5% (3/26) feeling it was extremely useful (Fig. 3). Residents embraced the idea of attending physicians providing further guidance as to how to utilize the provided resources, with 76.9% (20/26) of residents believing that such an effort would be “probably” or “definitely” helpful. No resident thought that providing this additional direction would be unhelpful.

When queried regarding their educational needs, the survey demonstrated more ambivalent results: 53.8% (14/26) of residents felt that their educational needs were probably or definitely being met, while 34.6% (9/26) were unsure their needs were met, and 11.5% (3/26) residents felt their educational needs were probably not being met. Despite the seemingly generalized acceptance of the creation of an online, supplementary curriculum, there was a greatest variation in resident response to the idea of utilizing pre- and posttest assessments to evaluate the efficacy of the provided online materials. A total of 26.9% of residents (7/26) felt that it would probably, or definitely, not be helpful. Alternatively, 42.3% (11/26) residents felt that it probably or definitely would be helpful while 30.8% (8/26) residents were unsure about the utility of pre- and post-assessments.

Lastly, the residents expressed a generalized approval and positive response to the residency program’s efforts to provide wellness materials addressing the unique challenges of being a healthcare provider during a pandemic. A total of 88.4% (23/26) residents were either somewhat or extremely satisfied with the program’s efforts with wellness. The changes in resident scheduling to promote resident safety were supported by most residents, with 80.8% (21/26) of residents reporting being somewhat, or extremely, satisfied with the changes. And, perhaps most importantly, 92.3% (24/26) of residents felt that the program’s response engendered a greater sense of belonging to their work community.

## Discussion

The COVID-19 pandemic has underscored the need for reliable and efficient communication within a residency program to allow for effective responses to rapidly evolving situations. Our study highlights the ability of Microsoft SharePoint to act as an intradepartmental distribution platform for educational materials, administrative decisions and scheduling, and wellness resources. Most residents surveyed felt that SharePoint was a useful addition to the residency program, and that its functionality would enhance their education by allowing greater input from educational leaders within the residency to curate their learning materials. Furthermore, its benefits extended to creating a greater sense of community and an environment of mutual support during a particularly tumultuous time. However, residents still expressed a general uncertainty that their educational needs were being met. Their response may reflect a broader understanding of “educational needs” to include not only quality didactic education but also quality readouts, patient and procedural experiences, and high case volumes, all of which had been negatively impacted by the COVID-19 pandemic. This also highlights that, while there has been a push for increased online materials and access by residents prior to social distancing, trainees still recognize the value of other aspects of the curriculum, like daily in-person educational lectures and "hot-seat" conferences.

In the broader context of medical and resident education, the incorporation of online resources into traditional curricula has only increased over time as learners have become accustomed to methods of learning outside of traditional books and lectures [12, 13]. Prior studies have documented the preference for rapidly accessed electronic sources to traditional textbooks or journal articles during day-to-day practice and the ability of online curricula to produce improvement over time [14–16]. However, as identified by Cook et al., there is much heterogeneity in the literature regarding what type of interventions constitute web-based learning initiatives and difficulties in comparing the effectiveness of web-based learning to traditional methods of instruction [17–19].

Given this heterogeneity, a platform for delivering web-based content for residency education must be adept at handling a variety of potential forms of education and be able to cross the many platforms of information technology. SharePoint accomplishes this by allowing a wide variety of media to be posted in the forms of PowerPoint lectures, word documents, PDFs, hyperlinks, discussion boards, and additional tools such as the “picture library” app to post visual content. SharePoint continues to evolve through the development of applications specific to SharePoint that can be downloaded from Microsoft and integrated into a given intranet site. Furthermore, SharePoint has Android and iPhone apps available in the respective Google Play and Apple App stores, facilitating ease of access on smart devices.

An enterprise-level solution such as SharePoint has many additional advantages for residency programs. It brings added security, as it is hosted on secure institutional servers and requires the same login credentials/dual-factor authentication as other secure sites within the institution. This security allows for the potential incorporation of internal educational materials that retain elements of personally identifiable healthcare information (PHI), if acceptable to local IT governance. Furthermore, individual files and parts of the site can have customized site permissions for different users, allowing editing capabilities or posting capabilities to be restricted to a subset of the members of the site. The management of site membership utilizing the university-wide directory within Microsoft Outlook was particularly important to us, as it allows for propagation of the website from year-to-year as residents graduate and new residents matriculate. Perhaps most importantly, it did not incur increased cost to the department as an enterprise-level solution.

Despite its likely widespread availability, and its positive attributes described above, we know of only one other residency program to report the utilization of SharePoint to accomplish its educational aims [20]. Our single-institution study demonstrated generalized acceptance of SharePoint as a distribution medium that must be replicated at other institutions to confirm its general utility to graduate medical education. Furthermore, our study is limited by lack of comparison with other online distribution platforms, utilization within the specialty of radiology alone, and lack of outcome measurements to evaluate the educational effectiveness of SharePoint. Further research into the cost-effectiveness of SharePoint at the enterprise level would provide a more nuanced understanding of how to incorporate it into organizations that do not currently possess institutional access to the Microsoft Office suite of programs. The COVID-19 pandemic may serve as a catalyst for broader acceptance of SharePoint as a distribution platform as all residencies adjust to educating while social distancing practices remain in force.

## Conclusions

Within a medium-sized residency composed of 31 residents, we have demonstrated the potential utility and generalized acceptance of Microsoft SharePoint as an online distribution platform for curricular and scheduling modifications, as well as the provision of wellness resources in response to the COVID-19 pandemic. Available as an enterprise-level solution at many large institutions, we believe Microsoft SharePoint can serve a similar function at many residency programs, both during the continued response to the COVID-19 pandemic and after.

## Supplementary Information

Below is the link to the electronic supplementary material.

Supplementary file1 (DOCX 33 KB)
