# Supplementary material for: The New Normal: Coronavirus Pandemic Response Utilizing Microsoft SharePoint
Source: J Digit Imaging. 2021 Mar 10;34(2):257–62. doi: 10.1007/s10278-021-00419-4 (PMC7946406; doi:10.1007/s10278-021-00419-4)
Supplement: Supplementary file 1 — Supplementary file1 (DOCX 33 KB) [file 10278_2021_419_MOESM1_ESM.docx]

**Supplemental Content**- Survey Instrument

Survey on education in UCD during Novel Corona virus pandemic 2020

Due to COVID-19 pandemic of 2020, all in person conferences were transitioned to videoconferences on March 13, 2020 in UCD Radiology. Please provide your opinion regarding this change:

For questions with scale of **1-5,** **1** = worst/lowest, **5** = best/highest

1. How would you rate **the overall effectiveness** of videoconferences for delivery of course material?
2. Success of technical delivery of material: (Scale of 1-5)

1 2 3 4 5

1. Learning experience:

1 2 3 4 5

1. With noon conferences being delivered via remote platform, are your educational needs being met? (Scale of 1-5)

1 2 3 4 5

1. How would you rate your level of engagement during videoconferences compared with in-person conferences?

1 = much lower, 2 = lower, 3 = same, 4 = higher, 5= much higher

1 2 3 4 5

1. How would you rate the ease of interaction/asking questions from the presenter during a videoconference compared with in-person conferences?

1 = much lower, 2 = lower, 3 = same, 4 = higher, 5= much higher

1 2 3 4 5

1. Would you like to see some use of remote conferencing in the future when social distancing guidelines are relaxed?

Yes No

1. How helpful has *Sharepoint* been during this pandemic? (Scale of 1-5)

1 2 3 4 5

1. As a result of the limited interaction between attending and trainees and our increased reliance on online resources for teaching, would guidelines regarding use of online resources for each rotation be helpful? (Scale of 1-5)

1 2 3 4 5

1. In terms of evaluating the effectiveness of online teaching material would an assessment (pre and post-test) given at the beginning and end of the rotation be helpful?

1 2 3 4 5

1. The resident schedule was reassessed and rearranged during the COVID-19 pandemic, for purposes of social distancing and in response to decreased volume of radiologic studies. A weekly schedule was generated by the PD based on section chiefs’ input about the sectional requirement. Sick days were handled separately. How satisfied are you with the changes in the schedule? (Scale of 1-5)

1 2 3 4 5

1. Several wellness initiatives – Meme contest, sharing of online wellness resources, meetings with PD are being carried out. How satisfied are you with the program’s efforts with wellness during this time? (Scale of 1-5)

1 2 3 4 5

1. Did the program’s efforts and response helped you make feel you belonged to your work community?

Yes No
